# Adherence to drug treatments and adjuvant barrier repair therapies are key factors for clinical improvement in mild to moderate acne: the ACTUO observational prospective multicenter cohort trial in 643 patients

**DOI:** 10.1186/s12895-015-0036-8

**Published:** 2015-09-11

**Authors:** Raúl de Lucas, Gerardo Moreno-Arias, Montserrat Perez-López, Ángel Vera-Casaño, Sonia Aladren, Massimo Milani, on behalf of ACTUO Investigators study group

**Affiliations:** Hospital Universitario La Paz, Madrid, Spain; Hospital Quirón Teknon, Barcelona, Spain; Clínica Dermatológica de Moragas, Barcelona, Spain; Hospital Carlos Haya,, Málaga, Spain; Isdin S.A. Medical Department, Provençals 33, Barcelona, Spain

**Keywords:** Acne vulgaris, Emollients, Observational study, Patient adherence, Topical administration

## Abstract

**Background:**

In acne, several studies report a poor adherence to treatments. We evaluate, in a real-life setting conditions, the impact of compliance to physician’s instructions, recommendations and adherence to the treatments on clinical outcome in patients with mild to moderate acne in an observational, non-interventional prospective study carried out in 72 Dermatologic Services in Spain (ACTUO Trial).

**Methods:**

Six-hundred-forty-three subjects were enrolled and 566 patients (88 %) completed the 3 study visits. Study aimed to evaluate the impact of adherence (assessed with ECOB scale) on clinical outcome, as well as how the use of specific adjuvant treatments (facial cleansing, emollient, moisturizing and lenitive specific topical products) influences treatment’s adherence and acne severity (0–5 points score). Recommendation of specific adjuvant skin barrier repair products was made in 85.2 %.

**Results:**

Overall, clinical improvement was observed throughout follow-up visits with an increased proportion of patients who reported reductions of ≥50 % on the total number of lesions (2 months: 25.2 %; 3 months: 57.6 %) and reductions of severity scores (2.5, 2.0 and 1.3 at 1, 2 and 3 months after treatment, respectively). Adherence to treatment was associated with a significant reduction on severity grading, a lower number of lesions and a higher proportion of patients with ≥50 % improvement.

**Conclusions:**

Good adherence to medication plus adherence to adjuvants was significantly associated with a higher clinical improvement unlike those that despite adherence with medication had a low adherence to adjuvants. A good adherence to adjuvant treatment was associated with improved adherence and better treatment outcomes in mild to moderate acne patients. (ISRCTN Registry: ISRCTN14257026).

## Background

Acne is a common skin chronic disease that very often requires prolonged treatments [1]. With a chronic course [2] and an episodic pattern of presentation [3], Acne vulgaris lesions occur mainly in exposed areas like the face and the presternal region. The effect of the scarring may be notable and the functional, social and emotional impact on the patient’s quality of life may be significant [4, 5]. Adherence to doctor prescriptions has a major impact on treatment outcome [6]. Several studies conducted over the past few years suggest that adherence to acne medications is particularly low [3, 7] and has been associated with inadequate response to therapy [8], especially when both topical and systemic treatments are prescribed to the same patient [9]. The treatment plan and the choice of specific active ingredients should take into account not only the individual characteristics of the patient and/or the disease but also their preferences and expectations with treatment as well as questions of convenience [10].The adverse effects of the various therapies available should also be borne in mind in order to increase adherence to the physician’s instructions and recommendations, thus promoting what are known as health-related behaviours [7]. Specific systemic and topical acne treatments are very often associated with local side effect such as dry, burning and itching sensations [7] hence poor tolerability further worsens compliance [11].^,^Based on the evidence and clinical experience obtained with topical treatments and their possible irritant effects [12], adjuvant skin barrier repair therapies such as specific emulsion and detergent products, hydrating and emollients are often prescribed in order to reduce these side effects, all in search of improving adherence to therapeutic strategies [13, 14]. Other factors such as a good and efficient physician-patient relationship are also crucial for improving adherence to therapeutic strategy [15] since this interaction includes specific roles and motivations [16] that might be essential to the healing of many patients, particularly so for patients with chronic disease or disease having a negative impact on quality of life and self-esteem such as acne [17]. Adherence with acne therapies has been evaluated mainly in subjects irrespective to grading severity of the condition (from mild to severe forms) [8, 18] but, so far, few data are available regarding this conduct in patients with mild to moderate forms. We evaluated in a real-life setting condition the impact of adherence to dermatologist instructions and recommendations and adherence to the treatments x(specific anti acne treatments and adjuvant therapies) on the clinical outcome in patients with mild to moderate acne. The participating physicians of the ACTUO trial were practicing experienced Spanish dermatologists working in hospital outpatient dermatology services.

## Methods

ACTUO was a multicenter, prospective, observational, epidemiological study of patients with mild to moderate acne treated at 72 dermatology services throughout Spain between October 2011 and November 2012. All study materials were evaluated and approved by a Clinical Research Ethics Committee before the study start. Approval (Ethics Committee of “Centro Medico Teknon” Barcelona Spain) was obtained August 1, 2011.

- ***Subjects***

A total of 643 subjects with mild to moderate acne, after they provided written informed consent, were enrolled in the study. Eligibility criteria were men and women with mild to moderate acne vulgaris eligible for a specific acne treatment (topical retinoid agents or antiseptics and/or systemic antibiotics) willing to participate in the study. The study was conducted at three visits carried out in a period of three months (V1: baseline, and two follow-up visits: V2 and V3). In all 3 visits, data on acne severity was recorded on the Case Report Form (CRF) by the dermatologist using the global ranking system of the FDA [19] (0: no lesions, 4: severe) and the total number of acne lesions. Acne severity was also evaluated by the patient. In addition, adherence to anti-acne treatments and adjuvant therapies was also assessed trough study.

b) ***Primary outcome***

The main objective of this analysis was to evaluate the impact of adherence to the treatments on the clinical outcome in patients with mild to moderate acne. A secondary outcome was to evaluate the impact of the use of specific adjuvant treatments (facial cleansing products, emollient moisturizing and lenitive specific topical cream products containing mainly rhamnosoft as emollient and antiflammatory agent) on adherence level and entity of the clinical outcome obtained. Acne severity was assessed at each visit with a 5-point score system (from 0 to 4), absolute count of lesions and percentage of patient reaching a ≥50 % reduction in lesion numbers. Adherence to treatment was evaluated at visit 2 and visit 3 by means of validated 4-item questionnaire (ECOB) with a dichotomous classification: good adherence (ECOB score = 4) and poor adherence (ECOB <4). Poor adherence to treatment was defined as a different to expected answer on the ECOB questionnaire. The four questions of ECOB were according to Pawin et al. [20].

The degree of adherence to the adjuvant measures prescribed by the doctor (application of cleansers and moisturizers for facial skin care) was assessed using the data provided by the patients attending all three study visits. It was considered that patients who followed all their doctor’s instructions and recommendations in at least three of the four assessments showed compliance with the medical advice. The effects of adherence to adjuvant treatment on the doctor’s and patient’s final assessments of acne severity was compared in patients "without /practically without lesions" (FDA scores = 0–1) and patients "with lesions" (FDA scores ≥ 2–4). The ACTUO study has evaluated also the impact of acne and acne treatments on quality of life evaluated by means of Cardiff Acne Disability Index [21] (CADI) and Dermatology Life Quality Index (DLQI) [22]. However these data would be presented elsewhere.

c) ***Statistical methods***

The FDA ratings made by the physician and patient after three months were grouped into two categories: presence of lesions (FDA ≥2-4), or absence or near absence of lesions (FDA = 0–1). Using the parameters *severity* and *total number of lesions*, improvement was assessed by classifying patients into two groups based on the reduction of acne severity (≥50 % improvement vs. improvement <50 %) and the percentages of reduction of comedones and papules/pustules. Statistical analysis was performed using the statistical software SPSS ver.19. Continuous variables were expressed as mean (SD). Categorical binary variables were expressed as proportions (%). The Mann–Whitney, Wilcoxon and the chi-square tests were used for inference statistical analysis tests. A multivariate logistics regression analysis was performed in order to assess the correlation between improvement of acne severity score, percentage of patients with 50 % or more in acne lesion number reduction, and the following variable: demographic data, adherence to specific treatments and to adjuvant treatments.

## Results

**Fig. 1:**
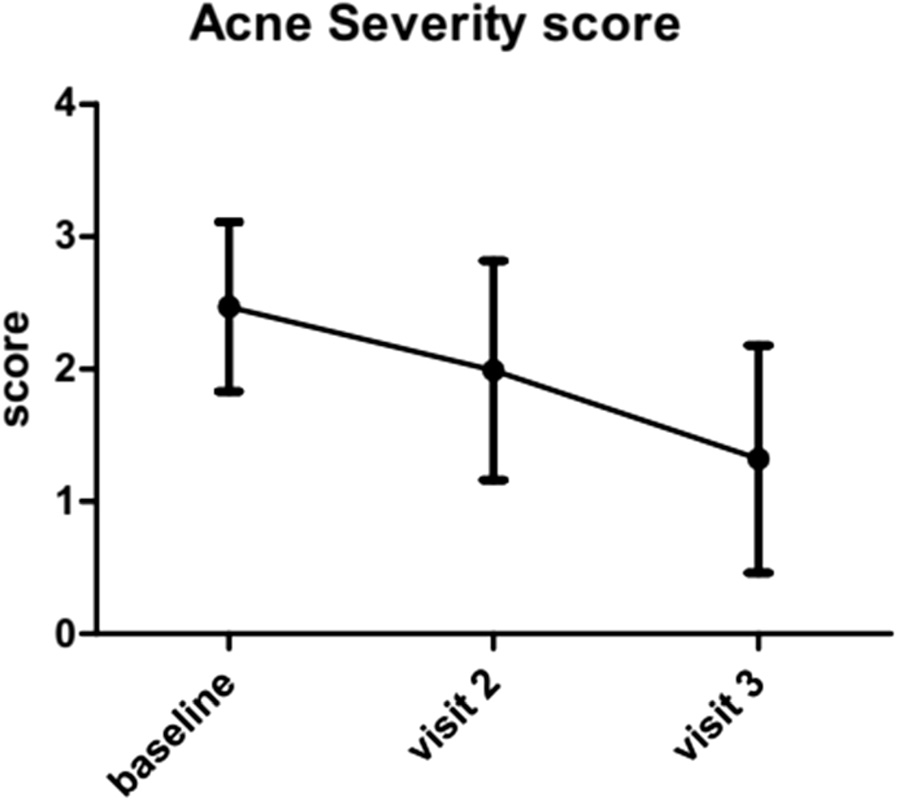
Evolution of acne severity score in the ACTUO study population as a whole

Table 1 gives information about patients’ characteristics at the time of enrollment. A total of 643 cases were enrolled. A total of 566 patients (88 %) completed the 3-visit study. Data are presented as per protocol analysis. At baseline, adjuvant products were prescribed in 83.8 % of the patients. The application frequency was 1.3/day. Prescription for specific adjuvant products was made for 85.2 % of the patients. Severity of acne assessed by the physician was 2.5 ± 0.6 and mean number of comedones was 18.9 ± 2. Severity acne score was significantly (*p* < 0.001) reduced in comparison with baseline after 1 month (2.0 ± 0.8) and after 3 months (1.3 ± 0.9). At the end of study period patients scoring 0 (complete cure of acne) were 17 %. In general, significant reductions in the severity scores vis-à-vis the baseline visit were observed in both doctor's and patients’ assessment (2.1 ± 0.9 at one month and 1.4 ± 0.9 at three months; *p* < 0.001). Figure 1 shows the evolution of acne severity score in the population as a whole. Percentage of patients showing at least 50 % of lesion reduction was 25.2 % at visit 2 and 57.6 % at visit 3.

**Table 1:** Sociodemographic data and acne severity at baseline visit

		n	mean (SD)	n (%)
Gender	Male	635	--	240 (37.8 %)
	Female			395 (62.2 %)
Age		617	21.3 (7.2)	
Study level	Primary school	632	--	24 (3.8 %)
	Secondary school			151 (23.9 %)
	Vocational training (school)			51 (8.1 %)
	Pre-university			188 (29.7 %)
	Vocational training (technical college)			30 (4.7 %)
	First cycle university studies			41 (6.5 %)
	Second cycle university studies			146 (23.1 %)
	Others			1 (0.2 %)
Physician’s acne evaluation	Without lesions	640	2.47 (0.64)	--
	Practically without lesions			32 (5.0 %)
	Mild			298 (46.6 %)
	Moderate			290 (45.3 %)
	Severe			20 (3.1 %)
Patient’s acne evaluation	Without lesions	594	2.10 (0.85)	16 (2.7 %)
	Practically without lesions			126 (21.2 %)
	Mild			245 (41.2 %)
	Moderate			195 (32.8 %)
	Severe			12 (2.0 %)
Lesion location^a^	Facial	637	--	622 (97.6 %)
	Back			212 (33.3 %)
	Shoulders			81 (12.7 %)
	Presternal area			73 (11.5 %)
	Others			2 (0.3 %)
Total number of lesions^a^(Percentages of 568 patients with lesions)	Comedones	546	18.9 (20.4)	532 (93.7 %)
	Papules/pustules	551	11.4 (10.2)	545 (96.0 %)
	Nodules	378	0.9 (1.7)	147 (25.9 %)
Recommendation of specific adjuvant products (facial soaps/cleansers and specific facial care products)	No	640	--	104 (16.3 %)
	Yes			536 (83.8 %)

^a^Non-exclusive categories

*N* number of patients, *SD* standard deviation; % = Percentage

**Fig. 2:**
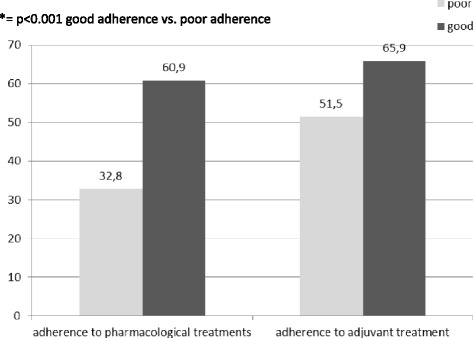
Patients with Acne improvement ≥50 % and regarding adherence to pharmacological and compliance with adjuvant treatments

According to the ECOB scores, good adherence to treatment was documented in 50.0 % of the patients at visit 2 and in 66.3 % at visit 3. Good adherence to treatment was associated with a significant (*p* < 0.05) acne improvement in comparison with poor adherence group at both control visits. At visit 3 acne severity score was 1.19 ± 0.8 in patients with good adherence to adjuvants vs. 1.4 ± 0.9 in poor adherence group. Good adherence to specific acne treatments (facial cleansers) was documented in 83.6 % at visit 2 and in 89.9 % at visit 3. At the end of study, patients with good adherence to treatment presented a significant improvement of acne both in term of acne severity score (1.2 vs. 1.7; *p* = 0.001) and regarding the proportion of patients whom reduction of lesions was ≥50 % (60.9 % vs. 32.8 %; *p* < 0.001) when compared to those with poor adherence (Fig. 2). In patients for whom the dermatologist had prescribed adjuvant therapies, a good adherence to this treatment was documented in 36.2 %. Adherence to adjuvant treatment improves acne thus it was associated with a significant reduction of score grading severity at visit 3 (1.2 vs. 1.4; *P* = 0.002), with a higher percentage of reduction of average lesions number vs. baseline (50.8 % vs. 43.7 %; *p* = 0.015) and with more patients obtaining a ≥50 % reduction in acne lesions’ number (65.9 % vs. 51.5 %; *p* = 0.003) in comparison with the low adherence group (Fig. 2). Adherence to adjuvant treatments was associated with a greater proportion of patients with a complete cure of acne (defined as no or very few acne lesions) at the final visit in comparison with poor-adherers (66.5 % vs. 52.6 %; *p* = 0.004). In particular, good adherence to adjuvant treatments improved the adherence to topical retinoid therapy therefore influencing clinical improvement of acne showing a higher proportion patients with adherence to treatment in adherers to adjuvants when compared to non-adherers (85.5 % vs. 70.7 %; *p* < 0.001). In addition, in patients with a good adherence toadjuvants were significantly more frequently considered with an ≥50 % improvement on clinical outcome in comparison with those that did not adhere to adjuvants (65.9 % vs. 51.5 %; *p* = 0.003).

Multiple logistic regression analysis was performed to identify factors influencing clinical improvement of acne. Adherence to specific treatments, adherence to adjuvant treatments and good adherence to treatment are significantly (*p* = 0.001) correlated with clinical improvement of acne. In accordance with previous studies, sex (women vs. men) and the fact to be accompanied (by parents/tutor or relative) or not at the medical visit are also independent factors affecting improvement clinical outcome of acne. Other analysis allowed observing an association between the patient’s assessment of quality of life based on the DLQI scale and the adherence to adjuvant treatment since rates of improvement on QoL were lower in non-compliers (43 % vs. 56.1 %, *p* = 0.007).

## Discussion

Low adherence to treatments is still a relevant problem in the treatment of mild to moderate acne [23]. The definition of strategies to improve adherence with both pharmacological and behavioural treatment remains a major challenge. Suboptimal medication adherence is one of the major reasons for treatment failure in subject with acne vulgaris [24]. Our findings reveal that dermatologists in Spain frequently prescribe specific adjuvant treatment for acne. They recommend the use of specific products including non-comedogenic soaps and moisturizers in over 80 % of acne patients. The synergy between the effects of drug treatment and adjuvant products can improve comfort during the various stages of treatment and can encourage patients to continue the application of the products. Adherence to adjuvant treatment was associated not only with a 2.4-fold increase in the probability of adherence to pharmacological treatment, but also with a significant reduction in acne severity, in the number of lesions as assessed by both the treating physician and the patient, and a higher percentage of patients whose severity improved by 50 % or more. In the ACTUO study low adherence to treatment was found in 50 % of the patients (at 1 month) and this result is in line with previous published data. Low adherence to adjuvant treatments was observed in up to 63.8 % of subjects after 3 months. In addition we have to consider that these data derived from a per protocol analysis, not including 12.4 % of patients not attending to control visits which therefore could be considered as”non-complier” by definition. In line with previous experiences [3], we have observed that adherence to pharmacological treatment is a key factor for the improvement of acne both in terms of reduction of severity grading, number of lesions and percentage of patients obtaining at least a 50 % acne improvement. Good adherence to adjuvant treatments also improves clinical evolution of acne with a greater reduction of severity of grading and number of lesions and with a significant greater percentage of patients obtaining a 50 % or more improvement of acne in comparison with patients with poor adherence to adjuvant treatments. Furthermore, patients with good adherence with drug and those with adherence to adjuvant treatments presented the highest percentage of patients with improvement of acne lesions ≥50 %. In addition, in the present study it was shown that adherence to adjuvant products is the second most important factor (just after adherence to drug treatment) influencing the degree of acne improvement. A good adherence to adjuvant treatment is associated with an improved adherence to acne treatments and with a better treatment outcome in mild to moderate acne patients. Some limitations should be taken in account in evaluating the results of our study. In particular the evaluation of compliance was based on self-reporting questionnaire. This approach however was used in other studies assessing the same outcome and the ECOB questionnaire is considered a validated tool [3, 18]. On the other hand, we believe that the sample size and the study design adopted, quite close to “real life” conditions of acne treatment strategies, could be considered as aspects increasing the external validity and the generalization of the observed results of the ACTUO.

## Conclusions

The ACTUO observational study results confirm that adherence to pharmacological treatment and adjuvant therapies are both key factors of acne improvement in terms of reduction of severity, number of lesions and percentage of patients with at least a 50 % acne improvement. In addition, adherence to adjuvant treatment with specific cleansers and moisturizers for patients with acne is the second most important factor in achieving symptom improvement, after completion of drug treatment. Good adherence to adjuvant treatment is associated with a 2.2-fold increase in the probability of adherence to topical pharmacological treatment and with significant reductions in the severity and number of acne lesions.

## Abbreviations

ECOB: Elaboration de un outil de evaluación de l’observance des traitaments medicamenteux
CRF: Case report form
FDA: Federal Drugs Administration
